# Diagnosis and treatment of renal neuroendocrine tumors: a case report and literature review

**DOI:** 10.3389/fonc.2025.1544525

**Published:** 2025-06-18

**Authors:** ShiHui Wang, JiongMing Li, JieShun Yang, Pei Li

**Affiliations:** The Second Affiliated Hospital of Kunming Medical University, Kunming, China

**Keywords:** renal neuroendocrine tumors, diagnosis, treatment, case report, literature review

## Abstract

Renal neuroendocrine tumors (NETs) are rare due to the absence of neuroendocrine cells in the kidney. Preoperative diagnosis is challenging often leading to misdiagnosis unnecessary nephrectomy. This study retrospectively analyzed three cases of renal NETs reviewing their diagnosis treatment processes. The first case involved a 46-year-old female presenting with lower abdominal pain diagnosed with a left renal NET (G2) post-surgery. The second case was a 56-year-old female with back pain diagnosed with a renal pelvic NET (G2) after laparoscopic nephroureterectomy. The third case was a 45-year-old female presenting with a palpable mass diagnosed with a right renal NET (G3) with liver metastases. All cases exhibited non-specific clinical presentations imaging findings highlighting the difficulty in preoperative diagnosis. Surgical resection was the primary treatment for non-metastatic cases while the metastatic case received a combination of surgical medical therapy. This study emphasizes the need for improved preoperative diagnostic methods to avoid aggressive surgical approaches to preserve renal units when possible. Further research is required to develop effective diagnostic tools treatment strategies for renal NETs.

## Introduction

Renal neuroendocrine tumors (NETs) are exceptionally rare due to the absence of neuroendocrine cells in the kidney (1). Neuroendocrine neoplasms (NENs) originate from neuroendocrine cells and peptidergic neurons, commonly found in the bronchial and gastrointestinal systems (2). Based on differentiation levels, they are classified into well-differentiated neuroendocrine tumors (NETs) and poorly differentiated neuroendocrine carcinomas (NECs) (3). Preoperative CT or MRI findings make it challenging to distinguish renal NETs from renal cell carcinoma and urothelial carcinoma, potentially leading to misdiagnosis and unnecessary nephrectomy (4, 5). This center retrospectively analyzed three cases of renal neuroendocrine tumors (NETs), reviewing their diagnosis and treatment processes. Combined with relevant literature, we analyze diagnostic and therapeutic deficiencies to share our experience in managing renal NETs. The patients provided informed consent for the publication of their clinical data.

## Case reports

### Case 1

**Figure 1:**
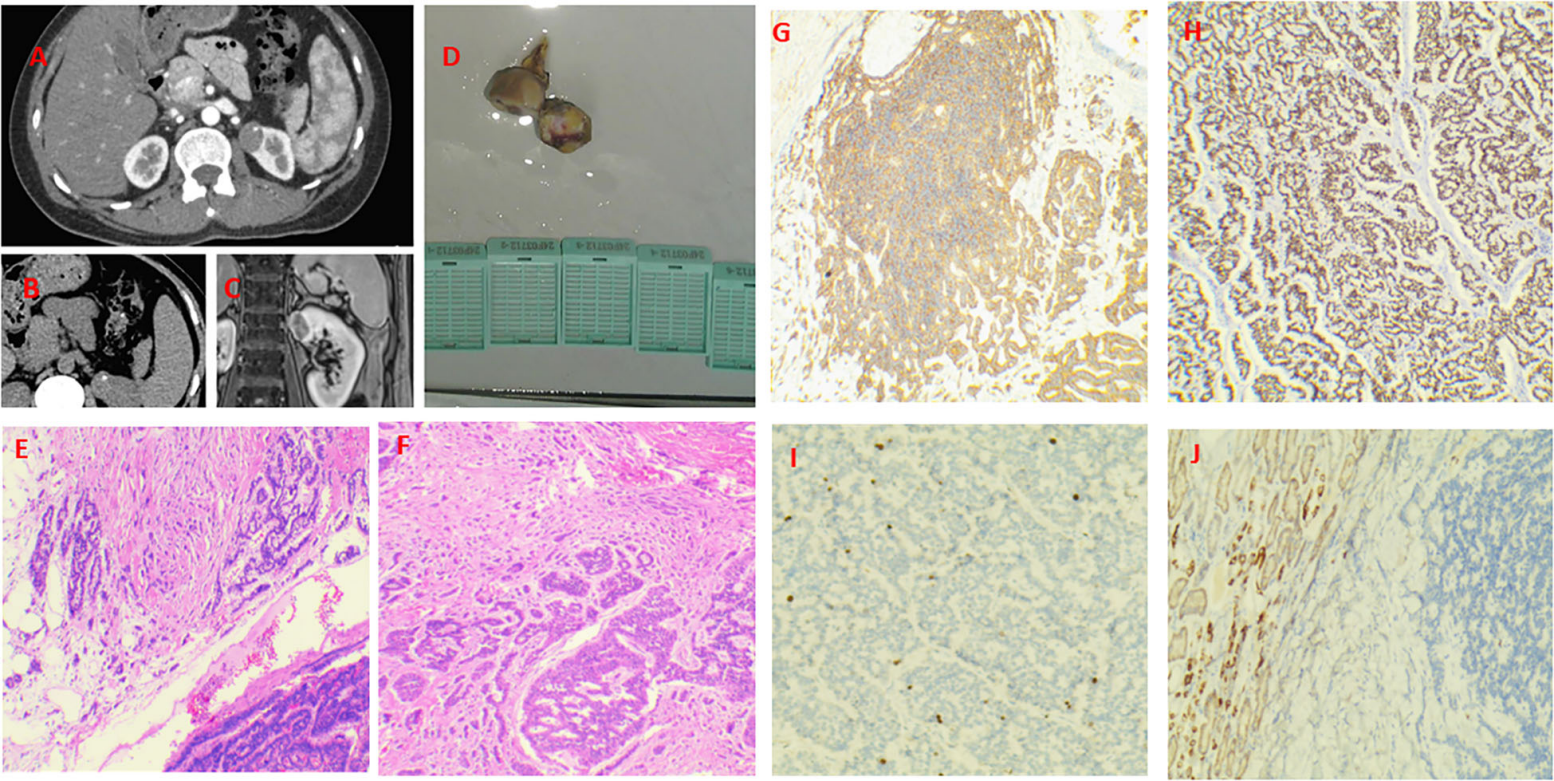
Case 1. Primary renal NET in a 46-year-old woman. A isodense nodule in the upper left kidney with multiple calcifications. There is a nodular mass with a mixed signal in the upper pole of the left kidney. It has clear margins and exhibits low - to - moderate enhancement during the cortical phase. **(A–C)** Gross specimen of the resected tumor (3×2 cm) with a gray-white to gray-brown cut surface and firm areas **(D)**. The tumor invades the perirenal fat **(E, F)**. Diffusely and strongly positive expression of Syn and INSM-1 **(G, H)**. The Ki-67 index was 5% **(I)**. The Ksp-Cadherin was negative **(J)**.

A 46-year-old female presented to hospital in January 2024 with lower abdominal pain. Imaging revealed a mass in the upper pole of the left kidney, suspected to be neoplastic. The patient reported persistent abdominal distension and pain, without fever, diarrhea, hematuria, or weight loss. Her medical history included depression with anxiety and lung nodule resection (benign on pathology) five years prior. Blood tests showed no significant biological abnormalities. Abdominal CT revealed an isodense nodule in the upper left kidney with multiple calcifications (**Figure 1A**), showing mild heterogeneous enhancement on contrast imaging (**Figure 1B**), measuring approximately 23×19mm. MRI showed a nodular mixed signal mass in the upper pole of the left kidney with clear boundaries and low to moderate enhancement in the cortical phase (**Figures 1C, D**), suggesting a renal mass suspicious for malignancy. Laparoscopic partial left nephrectomy was performed under general anesthesia. During the operation, a tumor was observed protruding from the surface of the kidney, with an intact capsule, measuring 3.5*2.2 cm. The cut surface was gray-white and gray-brown, with some areas being hard. Under the optical microscope, tumor cells were arranged in cords, irregular glandular shapes, or rings, with mitoses <2/10 high-power fields. Immunohistochemical staining revealed diffuse and strong positivity for insulinoma-associated protein 1( INSM-1), synaptophysin (Syn), and neural cell adhesion molecule (CD56), while chromogranin A (CgA) was negative. Markers for renal cell carcinoma (RCC ), kidney-specific cadherin (Ksp-Cadherin) and mast cell growth factor (CD117) were also negative, with a Ki-67 index of 5% (**Figures 1E–J**) The final diagnosis was left renal neuroendocrine tumor (NET G2). One month post-surgery, PET/CT showed no local recurrence or distant metastasis. So far, there have been no signs of recurrence or metastasis.

### Case 2

**Figure 2:**
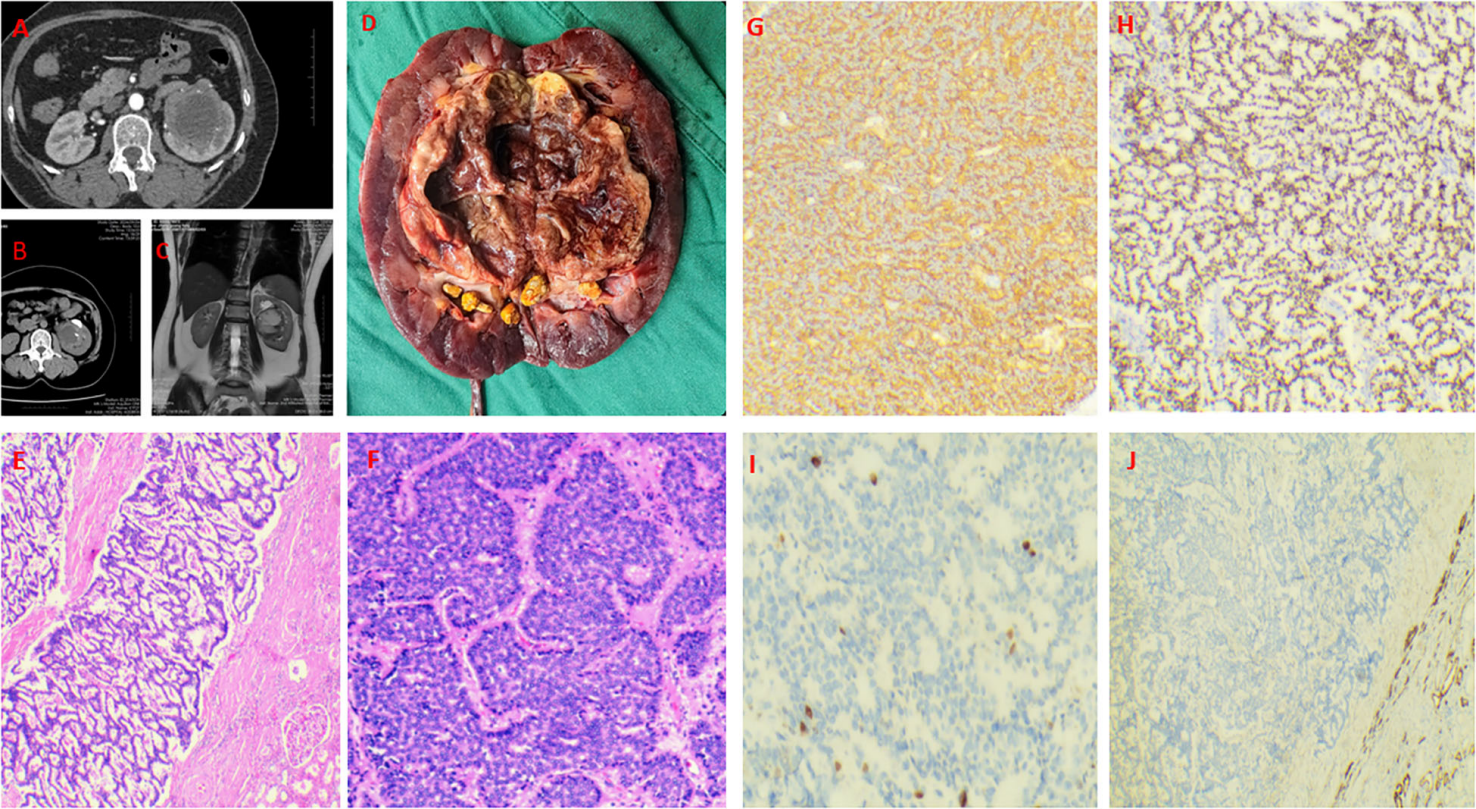
Case 2. Primary renal NET in a 56-year-old woman. A mixed-density mass in the left renal pelvis with heterogeneous enhancement of solid components and multiple stones in the collecting system **(A, B)**. A mixed signal mass in the left renal pelvis and hilum area with heterogeneous enhancement **(C)**. Gross specimen (7×6×3 cm) showing a cystic-solid mass with dark red fluid and necrotic debris **(D)**. Tumor cells are cribriform and irregularly glandular **(E, F)**. Diffusely and strongly positive expression of Syn and INSM-1 **(G, H)**. The Ki-67 index was 5% **(I)**. The CK7 was negative **(J)**.

A 56-year-old female presented to a local hospital in September 2024 with back pain. Retrograde urography suggested a left renal pelvic mass, suspicious for malignancy. Medical and family history were unremarkable, with no abnormal physical signs. Enhanced CT showed a mixed-density mass in the left renal pelvis with heterogeneous enhancement of solid components (**Figures 2A, B**), measuring approximately 71×63mm. MRI revealed a mixed signal mass in the left renal pelvis and hilum area with heterogeneous enhancement (**Figure 2C**), suggesting a renal pelvic mass suspicious for malignancy. Other examinations showed no abnormalities or metastases. Laparoscopic left nephroureterectomy was performed under general anesthesia. During the operation, it was found that the tumor was located in the left renal pelvis and hilar region, without invasion of blood vessels and lymphatic vessels. Upon incision, it was cystic - solid in nature, with a size of about 7 * 6 * 3 cm. The cut surface was gray - white and gray - red, soft in texture, with dark red fluid and necrotic materials flowing out, and there were multiple stones in the collecting system (**Figure 2D**). No invasion of the renal parenchyma was observed, and it had not broken through the renal capsule. Under the optical microscope, tumor cells were arranged in a sieve - like, irregular glandular or ring - like pattern. The cells were round to oval, with indistinct nucleoli, scant cytoplasm, and mitoses < 4/10 high - power fields. Immunohistochemical staining revealed diffuse and strong positivity for INSM-1, Syn, and CD56, while CgA was negative. Markers for RCC,Ksp-Cadherin, P40 and CK7 were also negative, with a Ki-67 index of 5% (**Figures 2E–J**).The diagnosis was renal neuroendocrine tumor (NET G2) originating from the renal pelvis, which is rare, with only 2 cases (4.3%) reported in a study of renal NET (6).

### Case 3

**Figure 3:**
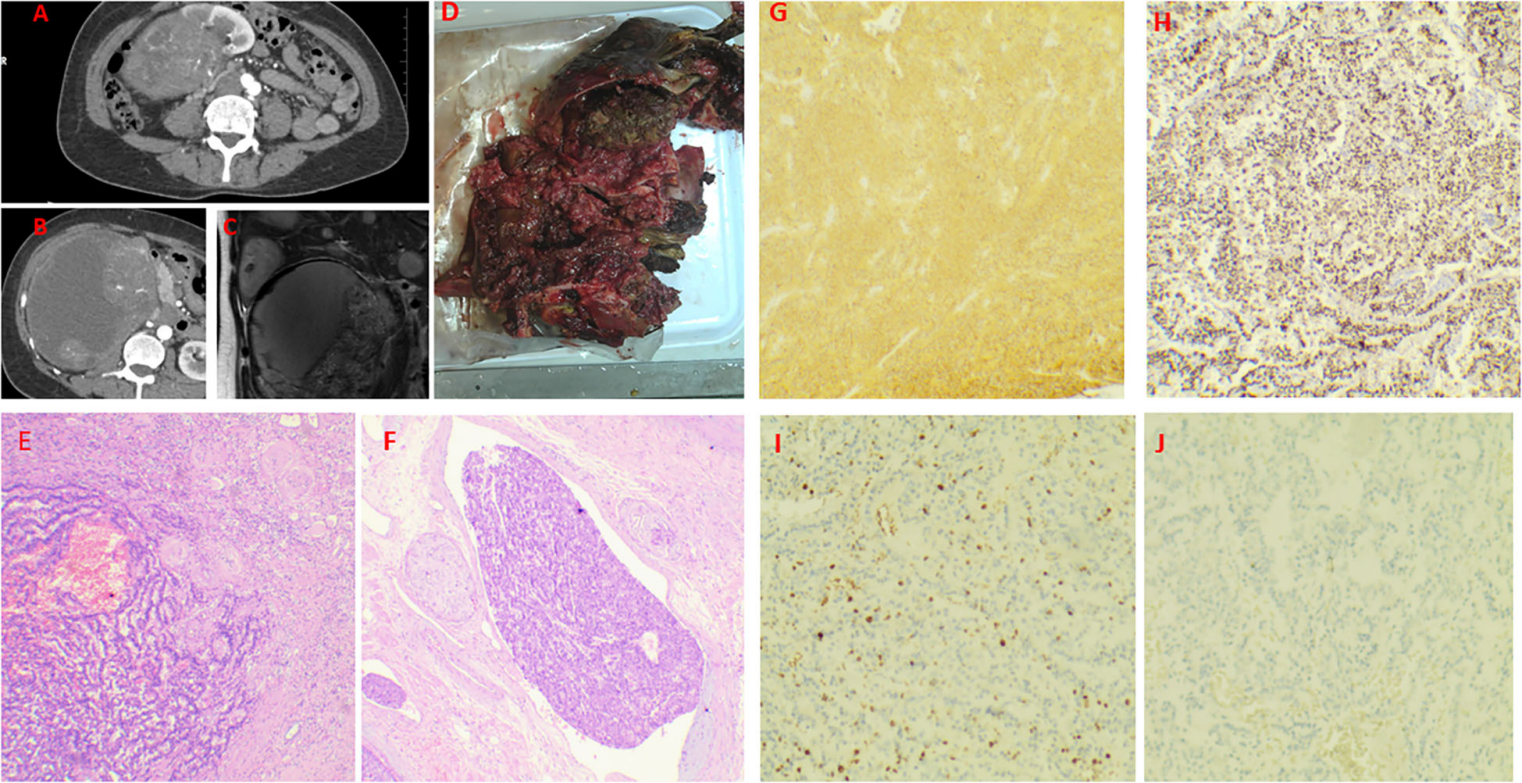
Case 3. Primary renal NET in a 45-year-old woman. a mixed-density mass in the right kidney measuring 15.3×15.6×12.6cm, with heterogeneous enhancement in early phase and decreased enhancement in late phase. Multiple nodular and patchy low-density lesions were noted in the liver with ring enhancement **(A–C)**. Gross specimen (18×12×15 cm) with a gray-white to gray-brown cut surface, focal hemorrhage, and necrosis **(D)**. The transition zone of the tumor, with the right side being the atrophic renal cortex **(E)**. Intravenous tumor thrombus **(F)**. Diffusely and strongly positive expression of Syn and INSM-1 **(G, H)**. The Ki-67 index was 30% **(I)**. Negative staining for RCC markers **(J)**.

A 45-year-old female was admitted to our hospital in January 2022 with a palpable hard mass in the right upper abdomen, without gross hematuria, facial flushing, or diarrhea. Medical and family history were unremarkable. Abdominal CT showed a mixed-density mass in the right kidney measuring 15.3×15.6×12.6cm, with heterogeneous enhancement in early phase and decreased enhancement in late phase. Multiple nodular and patchy low-density lesions were noted in the liver with ring enhancement (**Figures 3A–C**), suggesting right renal mass with multiple liver metastases. Open right nephrectomy, partial hepatectomy, and radiofrequency ablation of liver tumors were performed. The surgical specimen showed a kidney measuring 18×12×15cm, with gray-white to gray-brown soft cut surface and areas of apparent hemorrhage and necrosis (**Figure 3D**). Under the microscope, tumor cells were seen to be arranged in irregular glandular, nest - like or ring - like patterns, with moderate cellular pleomorphism. There was interstitial fibrous tissue hyperplasia accompanied by hyalinization, vascular hyperplasia, dilation and congestion, and patchy necrosis (accounting for about 5%) within the tumor. Immunohistochemical staining revealed diffuse and strong positivity for INSM-1, Syn, and CD56, while CgA was negative. Markers for RCC,Ksp-Cadherin and CD117 were also negative, with a Ki-67 index of 5% (**Figures 3E–J**). The diagnosis was renal neuroendocrine tumor (NET G3). The partial hepatectomy specimen reported a neuroendocrine tumor (NET G3), which was consistent with metastasis. Partial hepatectomy specimen confirmed metastatic NET. Post-operatively, the patient received regular antineoplastic therapy with capecitabine + temozolomide + octreotide LAR, and underwent three sessions of super-selective transcatheter hepatic arterial embolization + chemotherapy infusion within two years post-surgery. Everolimus was added after one year. The patient has survived with disease for over 30 months.

## Discussion

Primary renal neuroendocrine neoplasms (PRNENs) are a rare entity within the urinary system, with research in this field rapidly expanding but still facing numerous challenges and unresolved questions. In 2016, the World Health Organization (WHO) classification of tumors of the urinary system and male genital organs categorized PRNENs into well-differentiated neuroendocrine tumors (NETs), large cell neuroendocrine carcinomas (LC-NEC), small cell neuroendocrine carcinomas (SC-NEC), and paragangliomas (7). These tumors demonstrate significant heterogeneity, with widely varying treatment approaches and prognoses (8, 9). NETs typically exhibit low malignancy, slow growth, and favorable prognosis, often presenting as benign tumors, although poorly differentiated NETs can develop distant metastases (10). Currently, diagnosis relies on postoperative pathology and immunohistochemistry, with preoperative misdiagnosis and overtreatment being common issues, potentially leading to unnecessary loss of renal units and excessive treatment (11, 12).

Neuroendocrine neoplasms (NENs) originate from neuroendocrine cells and peptidergic neurons, but the pathogenesis of renal NENs remains unclear since neuroendocrine cells are typically absent in adult renal parenchyma (6, 8, 9). Several hypotheses regarding their origin include: 1) Metaplasia of the urothelial cells in the renal pelvis or calyces due to chronic inflammation (13); 2) Metastases from undiscovered primary tumors (5); 3) During embryonic development, neural crest cells may become trapped within the kidneys, possessing the potential to differentiate into neuroendocrine cells (14); 4) activation of shared genetic sequences for neuroendocrine programming in multipotent primitive stem cells (9); 5) concurrent congenital renal abnormalities (14). Most affected kidneys show evidence of chronic pyelonephritis, pyelitis, or kidney stones. The predominant location of NENs in horseshoe kidneys near the isthmus strongly supports anatomical malformation and metanephric cell migration as key mechanisms in renal NEN development (15). Most renal NENs are well-differentiated NETs with favorable prognosis (14, 16). We believe that during diagnosis and surgical planning, maximum preservation of renal units should be prioritized.

Renal NETs lack characteristic clinical presentations, with main reported symptoms including back pain, abdominal distension, abdominal mass, and hematuria. As neuroendocrine tumors, they have the potential to secrete bioactive substances, though carcinoid syndrome is reported in only 12.7% of patients (6). The most common neuroendocrine syndrome is carcinoid syndrome, primarily caused by excessive serotonin release (17), followed by glucagon-induced constipation, and Zollinger-Ellison, Verner-Morrison, and Cushing syndromes (9). When encountering patients with renal masses accompanied by these symptoms, renal NET should be included in the differential diagnosis. Clinically, we can use urinary serotonin testing and serum chromogranin (CgA) measurement. Traditional imaging methods like CT and MRI cannot definitively distinguish renal NETs from other kidney tumors, though they tend to show weak or poor enhancement on contrast CT, which can be a characteristic imaging feature for differentiation from other renal cancers (5, 6, 9). Neuroendocrine tumors typically show high somatostatin receptor (SSTR) expression, with over 85% of tumors having high-affinity receptors for somatostatin (18). Octreotide, a somatostatin analog (SSA) agonist, can be radiolabeled for NET diagnostic imaging (SRS) through somatostatin receptor scintigraphy. Radiolabeled somatostatin analogs used in peptide receptor radionuclide therapy (PRRT) represent a successful example of “theranostics” (2). The main limitation of this method is that normal kidney uptake of tracers may mask suspicious lesions (19), and it is primarily available only in research medical centers, making it difficult to implement in other facilities, which contributes to the challenges in preoperative diagnosis of NETs. Diagnostic biopsy is another method for preoperative diagnosis (20), but since kidney tumor guidelines do not recommend biopsy, clinicians face difficulties in making this decision, and specific evidence-based medical evidence requires verification through clinical trials.

In recent years, new diagnostic methods have emerged, particularly molecular imaging, with 68Gallium (Ga)-DOTATATE positron emission tomography (PET/CT) imaging now validated and routinely used. These studies demonstrate high sensitivity/specificity for detecting SSTR-positive NET location and extent. Additionally, 18F-fluorodeoxyglucose (FDG) PET is increasingly used in combination with 68Ga-DOTATATE for diagnosing aggressive (high-grade) tumors (21). This method can provide guidance for preoperative diagnosis and surgical treatment planning, though the preparation of radionuclide-labeled tracers remains a bottleneck in implementing this approach.

NET diagnosis primarily relies on pathology and immunohistochemical analysis. Most NETs are well-circumscribed solid masses, typically appearing gray-white or gray-brown on sectioning. Hemorrhage and necrosis are rare. In current studies, tumor cells are arranged in cord-like, trabecular, and small beam structures with abundant blood sinusoids. Cells are round or polygonal with eosinophilic cytoplasm and indistinct boundaries, round nuclei with uneven chromatin granules, mitoses, and rare necrosis. NETs specifically express neuroendocrine markers such as Syn, INSM-1,CD56 and CgA. These markers show high specificity and sensitivity for diagnosis. Studies have found Syn to have higher sensitivity than CgA (22).This study excluded the possibility of classical subtypes of renal cell carcinoma (RCC), such as clear cell carcinoma, papillary carcinoma, and chromophobe carcinoma, by negative expression of RCC markers including PAX-8, PAX-2, CD10, and CD116. Additionally, the absence of CD117 positivity, a characteristic of chromophobe RCC, was confirmed. In three cases, diffuse strong positivity for Syn, INSM-1, and CD56 clearly indicated neuroendocrine differentiation, establishing the diagnosis of primary renal neuroendocrine tumor.

Prognosis correlates with tumor stage and size, with adverse factors including age >40 years, tumor size >4 cm, purely solid morphology, mitotic rate higher than 1/10 HPF, Ki-67 index greater than 30%, metastasis at initial diagnosis, and tumor extension throughout the entire renal capsule (6, 8). Like other renal tumors, the metastatic rate of renal NETs directly correlates with tumor size. While renal NETs grow slowly in the retroperitoneal space with subtle clinical symptoms, their deep retroperitoneal location makes them difficult to detect, often resulting in large tumor volumes at initial diagnosis due to prolonged growth periods. Therefore, although primary renal NETs generally show less aggressive biological behavior than RCC, metastases are common (8). Studies report that 45.6% of patients present with metastases at initial diagnosis, and 59% of tumors larger than 4 cm develop metastases, making it essential to confirm the presence of distant metastases preoperatively (6). In our third case, liver metastases were present, attributable to prolonged tumor growth, large volume, and postoperative immunohistochemical Ki-67 index of 30%, consistent with previous research findings. This patient has survived with disease for over 30 months.

Surgical resection is the preferred treatment for non-metastatic patients. However, in cases with metastases, whether preoperative or postoperative, there are currently no guidelines directing systemic treatment for renal NETs. Everolimus is a key therapeutic agent for advanced NETs, usable as second-line treatment after SSA failure or third-line treatment after PRRT failure (23). Lymph nodes are the most common metastatic site for renal NETs, and the surgical approach for lymph node metastases is radical nephrectomy with lymph node dissection. Romero et al. reported that approximately 50% of renal NET patients with lymph node metastases who underwent radical nephrectomy + perirenal lymph node dissection showed no recurrence or metastasis after a mean follow-up of 43 months (6). However, for patients with tumors confined to the kidney without metastases, there is no consensus on choosing between radical nephrectomy, partial nephrectomy, or minimally invasive treatments like radiofrequency ablation. Some patients show no recurrence after partial nephrectomy during long-term follow-up, while others develop distant metastases even after radical nephrectomy. Therefore, the management of renal NETs remains controversial. Current research suggests that renal NETs are benign tumors, with partial nephrectomy or tumor radiofrequency ablation as the gold standard (14, 16). However, due to their rarity and similarity to other tumors, routine examination methods make preoperative diagnosis difficult. If we could complete diagnosis preoperatively, determining tumor grade and differentiation, and choose surgical methods that preserve renal units when possible, multiple studies have demonstrated that patients receiving nephron-sparing surgery have lower rates of postoperative chronic kidney disease (CKD) than those receiving radical resection, and preservation of renal function can improve and reduce overall mortality (24, 25). Cryoablation is also a gold standard surgical approach for NETs (14), and for patients with confirmed preoperative diagnosis, this approach represents a treatment modality that reduces medical expenses, alleviates patient suffering, and offers favorable prognosis.

NETs show lower malignancy and favorable prognosis, with surgical resection being the first choice for non-metastatic patients. However, some patients may still experience recurrence and metastasis after surgery, requiring close follow-up. The disease shows high heterogeneity and uncertain natural course, with studies showing that some renal NETs patients may develop systemic multiple metastases years after nephrectomy (8); therefore, even with well-differentiated, low-grade tumors or early clinical stage, patients require lifelong follow-up every three months.

In our three clinical cases, preoperative diagnoses were incorrect, and surgical plans were formulated according to renal malignancy protocols. Based on tumor size, location, and presence of systemic metastases, partial nephrectomy, radical nephrectomy, and radical nephrectomy + partial hepatectomy + hepatic radiofrequency ablation were performed respectively. However, postoperative pathology revealing primary renal neuroendocrine tumors has brought renewed attention to this disease. Renal NETs are rare, with non-specific clinical and imaging presentations, making them easily misdiagnosed as renal cancer or renal pelvic cancer, leading to aggressive surgical approaches. If preoperative diagnosis could be achieved through various methods such as PET/CT, radionuclide imaging, biopsy, and MDT multidisciplinary consultation, confirming the tumor as renal NETs, choosing nephron-sparing treatment modalities when possible would greatly benefit patients while improving clinical decision-making in the diagnosis and treatment of renal tumors.

## Limitations of the study and future directions

This study’s limitations include a small sample size (3 renal NETs cases), limiting generalizability. Heterogeneous treatments may obscure outcome differences. Follow-up duration remains insufficient to assess long-term recurrence. Future multicenter prospective studies with expanded cohorts, standardized methods, and integrated multi-omics analyses are critical to refine diagnostic/therapeutic strategies and advance precision medicine for these rare tumors.

## Data Availability

The original contributions presented in the study are included in the article/supplementary material. Further inquiries can be directed to the corresponding author.
